# Integrated Serum and Spatial Metabolomics Reveals Compartmentalized Metabolic Reprogramming and Ferroptosis Susceptibility in Nasopharyngeal Carcinoma

**DOI:** 10.3390/metabo16100783

**Published:** 2026-10-16

**Authors:** Huiling Hong, Yusong Zhang, Linhui Yan, Ting Xu, Yiying Xu, Zhaodong Fei, Jiyang Dong, Chuanben Chen

**Affiliations:** 1 Department of Radiation Oncology, Clinical Oncology School of Fujian Medical University, Fujian Cancer Hospital, NHC Key Laboratory of Cancer Metabolism, Fuma Road, Fuzhou 350014, China; hhl@fjmu.edu.cn (H.H.); yanlinghui@fjmu.edu.cn (L.Y.); xuting@fjzlhospital.cn (T.X.); xuyiying@fjzlhospital.cn (Y.X.); feizhaodong@fjmu.edu.cn (Z.F.); 2 Department of Electronic Science, National Institute for Data Science in Health and Medicine, Xiamen University, Xiamen 361005, China; zhangyusong@stu.xmu.edu.cn

**Keywords:** nasopharyngeal carcinoma, spatial metabolomics, mass spectrometry imaging, metabolomics, metabolic reprogramming, ferroptosis, biomarker, cysteine

## Abstract

Background: Nasopharyngeal carcinoma (NPC) is a malignancy highly prevalent in southern China. Metabolic reprogramming is a hallmark of cancer, yet the interplay between the systemic and local metabolic landscapes of NPC remains poorly characterized. This study aimed to identify serum metabolic biomarkers and characterize local tissue metabolic alterations through integrated metabolomics. Methods: We conducted a dual-compartment metabolomic investigation. Untargeted serum metabolomics was performed on 80 NPC patients and 35 healthy controls. Air flow-assisted desorption electrospray ionization mass spectrometry imaging (AFADESI-MSI) was applied to nasopharyngeal tissues (3 NPC vs. 3 chronically inflamed tissues). Cross-platform integration was performed to identify overlapping metabolic signatures, followed by least absolute shrinkage and selection operator (LASSO) regression for feature selection and logistic regression for biomarker panel construction. Results: Serum metabolomics revealed systemic perturbations involving amino acid and propanoate metabolism. Spatial metabolomics identified glycerophospholipid metabolism as the most significantly altered pathway in NPC tissues, with markedly reduced phosphatidylserine in tumor regions, and the ferroptosis pathway was significantly enriched. Cross-omics integration identified 8 overlapping metabolites, of which 5 were mapped to the biosynthesis of unsaturated fatty acids. Cysteine demonstrated excellent diagnostic performance (area under the receiver operating characteristic curve (AUC) = 0.903). A 5-metabolite combined model achieved an AUC of 0.904 (95% confidence interval (CI): 0.892–0.916). Conclusions: NPC exhibits compartmentalized metabolic reprogramming characterized by systemic amino acid and propanoate perturbations alongside local glycerophospholipid remodeling and ferroptosis susceptibility. The identified 5-metabolite serum panel shows considerable potential as a non-invasive diagnostic tool for NPC.

## 1. Introduction

Nasopharyngeal carcinoma (NPC) is a malignant tumor arising from the epithelial cells of the nasopharynx, characterized by marked geographic heterogeneity, with a high prevalence in Southern China, Southeast Asia, and South Asia [1]. The advent of multimodal therapeutic strategies made it possible for the 5-year survival rate for non-metastatic NPC to reach up to 80% [2]; however, a subset of patients still experience disease progression or recurrence. Treatment options for patients with recurrent or metastatic NPC remain limited, and chemotherapy resistance poses a formidable clinical challenge [3]. Therefore, elucidation of the molecular mechanisms underlying NPC pathogenesis, identification of novel diagnostic biomarkers, and discovery of therapeutic targets hold substantial clinical significance.

Metabolic reprogramming has been recognized as one of the core hallmarks of malignant tumors, in which cancer cells rewire metabolic pathways to meet the energy and biosynthetic demands required for rapid proliferation and survival [4]. Metabolomics, as an important branch of systems biology, enables high-throughput detection of small-molecule metabolites in biological samples, providing a comprehensive snapshot of the metabolic state of the organism or tissue [5]. In NPC, Epstein–Barr virus (EBV) infection, hypoxia, and the tumor microenvironment collectively drive significant alterations in glycolysis, nucleotide synthesis, and lipid metabolism [3,6,7]. Lipid metabolic reprogramming is recognized as one of the most prominent metabolic abnormalities in cancer, and fatty acid metabolism emerges as a potential therapeutic target for NPC [8]. Studies have also revealed that NPC-associated metabolic reprogramming can promote tumor progression and immune suppression through intercellular communication [9].

The metabolic microenvironment of tumor tissues exhibits significant spatial heterogeneity. The tumor microenvironment comprises diverse cell types. Within it, cancer cells acquire growth advantages through metabolic reprogramming, which is profoundly influenced by local factors such as hypoxia and nutrient deprivation [10]. However, conventional metabolomics based on tissue homogenization destroys this spatial architecture during sample preparation and yields only averaged metabolite profiles [11]. Consequently, the in situ spatial distribution of metabolites across tumor and stromal regions is lost, limiting precise characterization of localized metabolic reprogramming. In recent years, advances in spatial metabolomics technologies, particularly mass spectrometry imaging (MSI), have enabled the simultaneous acquisition of qualitative, quantitative, and spatial distribution data of metabolites while preserving tissue integrity, thereby offering a robust platform for dissecting the metabolic heterogeneity of the tumor microenvironment [12]. In head and neck cancers, spatial metabolomics has been preliminarily applied to characterize metabolic reprogramming features of cancer cells [13]. A recent study has integrated spatial metabolomics with spatial transcriptomics in NPC, revealing that reprogramming of lipid, branched-chain amino acid, and glutamine metabolism is closely associated with therapeutic response and programmed cell death protein 1 (PD-1) expression [14].

However, most current metabolomics studies are largely restricted to a single type of biological sample, thereby precluding systematic integrative analyses that could link systemic and local metabolic alterations. Key questions—such as how tumor-derived metabolites enter the circulation and to what extent serum metabolic profiles mirror the metabolic status of tumor tissues—remain largely unexplored. In this study, we integrated serum untargeted metabolomics with air flow-assisted desorption electrospray ionization mass spectrometry imaging (AFADESI-MSI) to systematically characterize the systemic-to-local metabolic landscape of NPC. Through cross-omics integrative analysis, we aimed to characterize metabolic alterations in the serum and tumor tissues of patients with NPC, identify shared metabolites and pathways across the two biological compartments, and evaluate the diagnostic performance of candidate circulating metabolites and a multimetabolite model.

## 2. Materials and Methods

### 2.1. Research Ethics and Sample Sources

The serum metabolomics cohort comprised 115 serum samples, including 80 from patients with NPC and 35 from healthy controls (HCs). The spatial metabolomics cohort comprised six independent biological specimens (three NPC and three chronically inflamed specimens(chronic inflammation, CI)), with three physical tissue sections analyzed for each specimen, all provided by Fujian Cancer Hospital. Given the limited number of independent biological specimens, the spatial metabolomics component was considered exploratory. Serum samples were also collected at the same institution. All NPC patients were pathologically confirmed, had complete clinical documentation, and provided serum specimens collected prior to treatment. Exclusion criteria included prior anti-tumor therapy and the presence of chronic metabolic diseases. Control subjects were healthy volunteers undergoing routine health checkups during the same period, matched to the NPC group for age and sex. The serum and tissue samples were derived from independent patient cohorts; therefore, no paired patient-level integration was performed. Plasma EBV DNA was quantified at the clinical laboratory of Fujian Cancer Hospital using real-time quantitative PCR according to the hospital’s standard protocol; EBV DNA positivity was defined as ≥500 copies/mL [15]. This study protocol was approved by the Ethics Committee of Fujian Cancer Hospital (approval number: K2024-299-01), and the requirement for written informed consent was waived by the ethics committee.

### 2.2. Serum Metabolomics

Peripheral venous blood was collected after an overnight fast, allowed to clot at room temperature for 30 min, and centrifuged at 1500× *g* for 5 min at 4 °C. Serum was snap-frozen in liquid nitrogen and stored at −80 °C. Metabolites were extracted using liquid chromatography–mass spectrometry (LC–MS) grade methanol, followed by two-channel chemical isotope labeling (CIL) for amine/phenol and carboxyl metabolites, respectively. Chromatographic separation was performed on an Agilent 1290 ultra-high-performance liquid chromatography (UHPLC) system coupled to a 6546 quadrupole time-of-flight (Q-TOF) mass spectrometer. Detailed reagent compositions, gradient programs, and mass spectrometry parameters are provided in Supplementary Methods S2.

Raw data were processed using IsoMS Pro (v1.3.2). Metabolites were retained if detected in ≥80% of samples within one group, normalized by total useful signal (TUS), and identified against the CIL in-house library and lipid library by matching accurate mass (±10 ppm) and retention time.

### 2.3. Spatial Metabolomics

Frozen tissue sections (12 μm) were prepared using a cryotome and mounted onto positively charged slides. AFADESI-MSI [16] was performed on a platform coupled to a Q-Exactive Orbitrap mass spectrometer, with data acquired in both positive- and negative-ion modes at a spatial resolution of 50 μm × 50 μm. Detailed embedding protocols, spray solvent compositions, and acquisition parameters are provided in Supplementary Methods S3.

Raw MSI data were converted to imzML format, imported into MSiToolbox for preprocessing (denoising, peak alignment, total ion current (TIC) normalization, and low-quality feature filtering), and annotated by matching to a candidate molecular library derived from liquid chromatography–tandem mass spectrometry (LC–MS/MS) analysis of homologous tissue samples (±10 ppm). Detailed preprocessing pipelines and annotation criteria are provided in Supplementary Methods S4.

### 2.4. Statistical Analysis

For spatial metabolomics, the biological tissue specimen was treated as the independent unit for biological level inference (*n* = 3 NPC and *n* = 3 chronic inflammation); MSI pixels were treated as spatial observations nested within physical tissue sections, and the three sections of each specimen as within-specimen repeated measurements. For the biological specimen-level sensitivity analysis, pixel intensities were averaged within each section and the section means were averaged with equal weight per specimen; group differences were then evaluated by a two-sided exact permutation test enumerating all 20 possible 3-versus-3 label assignments, followed by Benjamini–Hochberg correction within each ion mode (minimum attainable exact *p* = 0.10). No feature reached significance after correction. The pixel-level Welch’s *t*-test was used exclusively as an exploratory feature-screening step and was not interpreted as biological replicate-level inference. Pixel-level UMAP and feature-level spatial screening were used for descriptive and exploratory analyses only and did not increase the biological sample size.

For spatial metabolomics, uniform manifold approximation and projection (UMAP) was applied for dimensionality reduction, and an exploratory pixel-level spatial screen was performed using Welch’s *t*-test with Benjamini–Hochberg correction (false discovery rate (FDR) < 0.05; fold change (FC) > 1.2 or FC < 0.83). Over-representation analysis (ORA) and gene set enrichment analysis (GSEA) were used for exploratory pathway characterization. For serum metabolomics, differential metabolites were identified using Welch’s *t*-test with variable importance in projection (VIP) > 1.0 from orthogonal partial least squares discriminant analysis (OPLS-DA), FDR < 0.05, and |FC| > 1.2. Quantitative enrichment analysis (QEA) and directional ORA were performed using MetaboAnalyst 5.0.

Cross-omics integration was performed by intersecting serum and tissue differential metabolites based on KEGG (Kyoto Encyclopedia of Genes and Genomes) IDs. Univariate and multivariate Firth penalized logistic regression were used to evaluate associations with NPC risk. To avoid information leakage, the entire feature-selection pipeline was embedded within an outer stratified 5-fold cross-validation (repeated with 10 random seeds). Within each training partition, serum differential metabolites were re-identified using training samples only (Welch’s *t*-test with Benjamini–Hochberg FDR < 0.05 and |fold change| > 1.2, together with VIP > 1.0 from OPLS-DA fitted on the training partition), intersected with the tissue-derived differential metabolite list (derived from the independent tissue cohort), and the resulting candidate metabolites were entered into a LASSO–logistic regression pipeline with an inner 5-fold cross-validation; each held-out fold was predicted by the model built on its corresponding training partition. Model performance was evaluated by the area under the receiver operating characteristic curve (AUC), sensitivity, specificity, accuracy, positive predictive value (PPV), negative predictive value (NPV), and F1 score. Detailed statistical parameters and validation procedures are provided in Supplementary Methods S5.

## 3. Results

### 3.1. Spatial Metabolomics Reveals the Characteristics of Local Metabolic Remodeling in Nasopharyngeal Carcinoma

To investigate local metabolic alterations in NPC tissues, AFADESI-MSI was performed on 6 tissue samples (3 NPC and 3 chronic inflammation; baseline characteristics are provided in Table 1).

**Figure 1:**
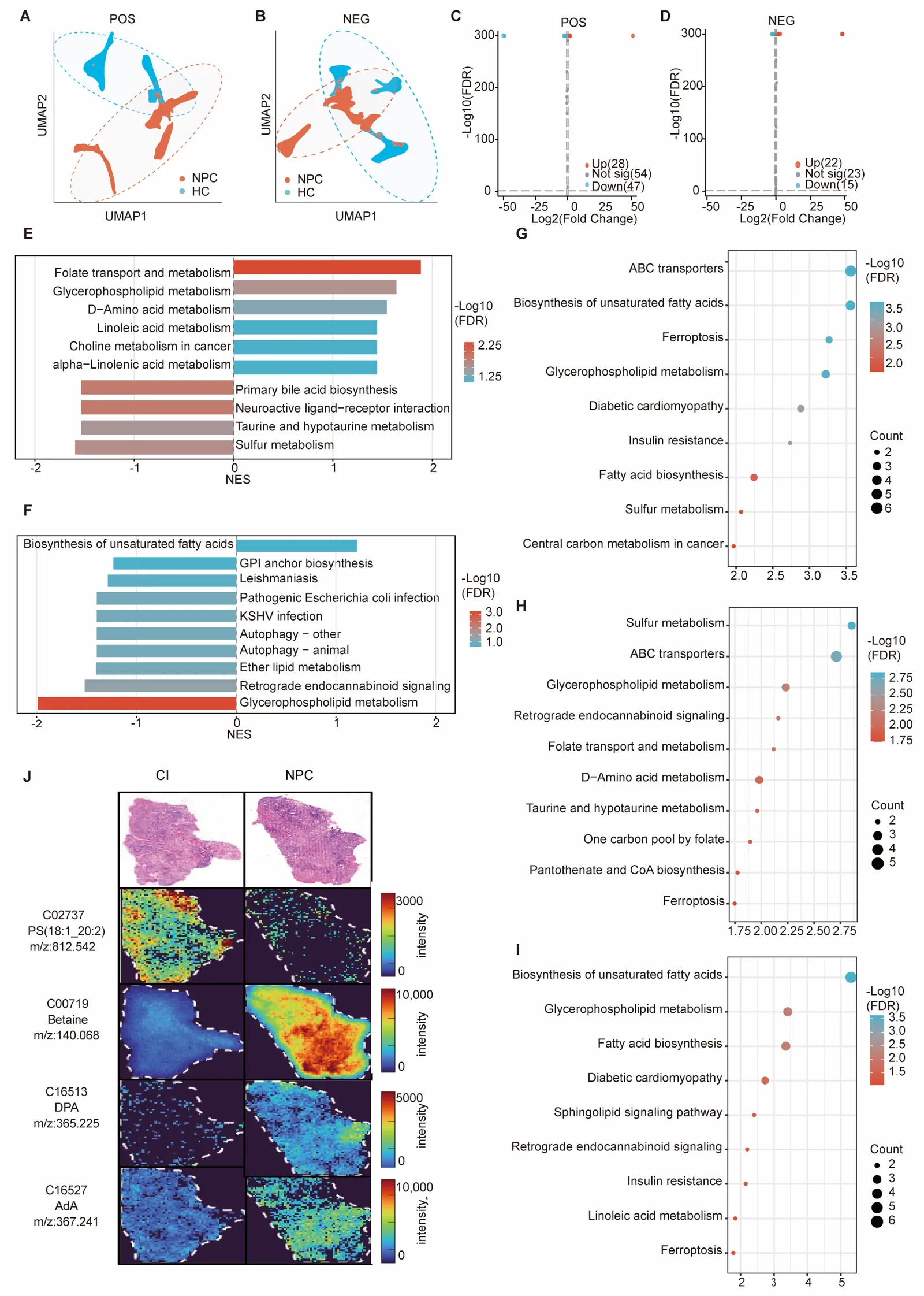
Spatial metabolomics reveals local metabolic reprogramming in nasopharyngeal carcinoma (NPC). (**A**,**B**) UMAP projections of MSI data in positive-ion (POS) and negative-ion (NEG) modes. (**C**,**D**) Volcano plots from the exploratory pixel-level spatial screen (FDR < 0.05; FC > 1.2 or FC < 0.83); red, higher pixel-level mean intensity in NPC; blue, lower pixel-level mean intensity in NPC. Dashed vertical lines indicate the fold-change thresholds (log_2_ FC = +0.26 for FC > 1.2, and log_2_ FC = −0.27 for FC < 0.83), and the dashed horizontal line indicates the significance threshold (FDR = 0.05; −log_10_(0.05) = 1.30).These thresholds do not denote biological specimen-level significance. (**E**,**F**) GSEA of pathways in POS and NEG modes; bars, normalized enrichment scores (NES). (**G**–**I**) Over-representation analysis (ORA); bubble size, number of hits; color intensity, FDR. (**J**) H&E staining (**top**) and corresponding AFADESI-MSI ion images of PS(18:1/20:2), betaine, DPA, and AdA (**below**); color scale, normalized ion intensity (blue, low; red, high). Panel (**J**) shows illustrative images from one NPC and one chronic-inflammation specimen, rather than a display of all six biological specimens. The quantitative sensitivity analysis used all six biological specimens as described in Section 2.4; none of the four metabolites was significant after BH correction. AFADESI-MSI, air flow-assisted desorption electrospray ionization mass spectrometry imaging; MSI, mass spectrometry imaging; UMAP, uniform manifold approximation and projection; FDR, false discovery rate; GSEA, gene set enrichment analysis; ORA, over-representation analysis; H&E, hematoxylin and eosin.

UMAP dimensionality reduction was applied to the normalized MSI feature matrix in both positive-ion (POS) and negative-ion (NEG) modes. In this exploratory analysis, two-dimensional UMAP projections revealed distinct clustering of NPC (red) and chronic inflammation (blue) groups in both POS (Figure 1A) and NEG (Figure 1B) modes, indicating pronounced metabolic divergence between the two groups.

In POS mode, 129 putatively annotated metabolites were detected. Using the exploratory pixel-level screening criteria of FDR < 0.05 and either FC > 1.2 or FC < 0.83, 75 candidate metabolites were retained, including 28 with higher and 47 with lower pixel-level mean intensities in NPC (Figure 1C). In NEG mode, 60 putatively annotated metabolites were detected, of which 37 met the same exploratory pixel-level screening criteria (22 with higher and 15 with lower pixel-level mean intensities in NPC) (Figure 1D). Exploratory candidates identified in POS mode were predominantly mapped to phospholipid and amino acid metabolism pathways, whereas NEG-mode features were primarily enriched in fatty acid metabolism. These screening counts do not represent specimen-level significant findings; no feature was significant after BH correction in the separate biological specimen-level sensitivity analysis.

To further characterize the directionality of metabolic pathway alterations, pre-ranked GSEA was performed based on log_2_FC-ranked lists of all annotated metabolites in POS and NEG modes separately, with enrichment strength quantified by the normalized enrichment score (NES). In POS mode, folate transport and metabolism (hsa04981) exhibited the strongest positive enrichment (NES = 1.88, FDR = 0.031), driven primarily by betaine (C00719) and phosphatidic acid (C00157) (Figure 1E). Additional pathways, including glycerophospholipid metabolism (NES = 1.64, FDR = 0.176), D-amino acid metabolism (NES = 1.54, FDR = 0.250), and biosynthesis of unsaturated fatty acids (NES ≈ 1.44), also showed positive enrichment trends. In NEG mode, glycerophospholipid metabolism (hsa00564) demonstrated the strongest negative enrichment (NES = −1.99, FDR = 0.0015), driven primarily by phosphatidylserine (C02737), phosphatidylglycerol (C00344), and phosphoethanolamine (C00350) (Figure 1F). Primary bile acid biosynthesis also showed a negative enrichment trend (NES = −1.53, FDR = 0.089).

Over-representation analysis (ORA) of the combined POS- and NEG-mode differential metabolites identified six significantly enriched KEGG pathways (FDR < 0.05): ABC transporters, biosynthesis of unsaturated fatty acids, ferroptosis, glycerophospholipid metabolism, diabetic cardiomyopathy, and insulin resistance (Figure 1G). Stratified ORA of POS-mode-specific differential metabolites further revealed significant enrichment of ABC transporters and sulfur metabolism (FDR = 0.0457), with folate/one-carbon metabolism-related pathways showing suggestive enrichment (Figure 1H). Conversely, NEG-mode-specific ORA identified significant enrichment of biosynthesis of unsaturated fatty acids (FDR = 0.00024) and glycerophospholipid metabolism (FDR = 0.0072) (Figure 1I).

To illustrate spatial localization, Figure 1J shows AFADESI-MSI ion images from one NPC specimen and one chronic-inflammation specimen; these images are illustrative rather than a display of all biological replicates. In the specimen-level sensitivity analysis across all six specimens, the group-mean directions were consistent with the displayed contrasts for PS(18:1/20:2), betaine, DPA, and AdA, although none remained significant after Benjamini–Hochberg correction. To spatially corroborate these pathway-level observations, ion images of representative metabolites were acquired (Figure 1J). Phosphatidylserine (PS(18:1/20:2); *m*/*z* 812.542, [M − H]^−^) exhibited markedly reduced ion intensity in tumor regions relative to chronic inflammation regions, whereas betaine (C00719), docosapentaenoic acid (DPA, C16513), and adrenic acid (AdA, C16527) all displayed elevated signal intensities in tumor regions.

### 3.2. Serum Metabolomics Reveals Systemic Metabolic Perturbations in NPC

To characterize systemic metabolic alterations in NPC, untargeted metabolomics was performed on 115 serum samples (80 NPC patients and 35 healthy controls; baseline characteristics are provided in Table 2).

**Figure 2:**
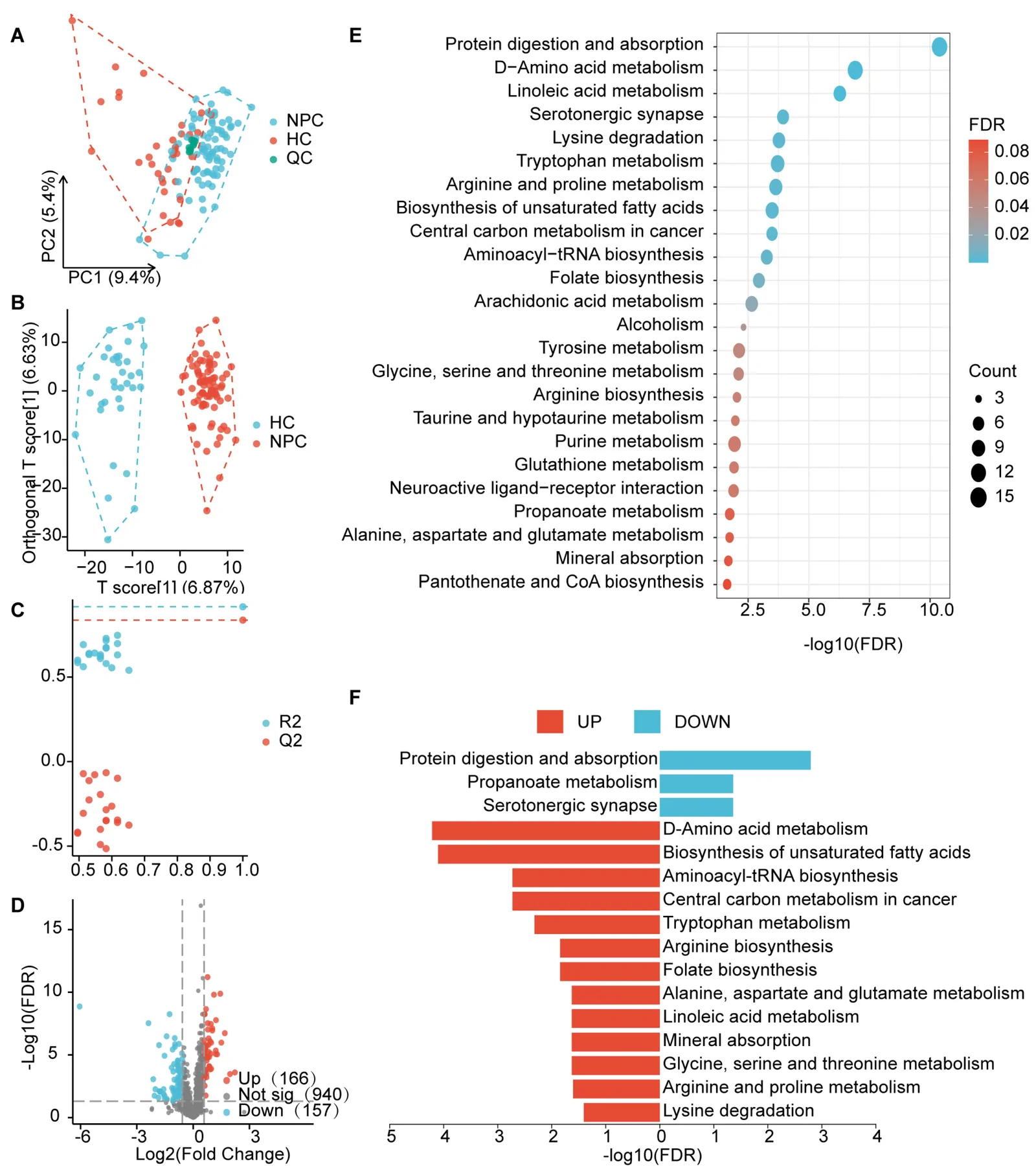
Serum metabolomics reveals systemic metabolic perturbations in NPC. (**A**) PCA score plot. (**B**) OPLS-DA score plot. (**C**) Permutation test of the OPLS-DA model (*n* = 200); R^2^Y = 0.916, Q^2^ = 0.837. (**D**) Volcano plot of differential metabolites (VIP > 1.0, FDR < 0.05, |FC| > 1.2); red, up-regulated; blue, down-regulated. Dashed vertical lines indicate the fold-change thresholds (log_2_ FC = ±0.26, corresponding to |FC| = 1.2), and the dashed horizontal line indicates the significance threshold (FDR = 0.05; −log_10_(0.05) = 1.30). (**E**) Quantitative enrichment analysis (QEA); bubble size, number of hits; color intensity, FDR. (**F**) Directional over-representation analysis (ORA); bar length, −log_10_(FDR); blue, down-regulated metabolites; red, up-regulated metabolites. NPC, nasopharyngeal carcinoma; PCA, principal component analysis; OPLS-DA, orthogonal partial least squares discriminant analysis; QEA, quantitative enrichment analysis; ORA, over-representation analysis; FDR, false discovery rate; FC, fold change; VIP, variable importance in projection.

Principal component analysis (PCA) revealed a discernible separation trend between the NPC and healthy control groups (Figure 2A). The OPLS-DA score plot further demonstrated clear separation between the two groups (Figure 2B). Permutation testing (*n* = 200) yielded goodness-of-fit and predictive parameters of R^2^Y = 0.916 and Q^2^ = 0.837, respectively, indicating acceptable model robustness and minimal overfitting (Figure 2C).

A total of 1263 metabolites were identified by LC–MS. Applying thresholds of VIP > 1.0, FDR < 0.05, and |FC| > 1.2, we identified 323 differential metabolites, comprising 166 up-regulated and 157 down-regulated features in NPC serum. Following KEGG annotation, 189 differential metabolites were subjected to pathway enrichment analysis (107 up-regulated and 82 down-regulated), with the overall distribution illustrated in the volcano plot (Figure 2D).

To systematically evaluate NPC-associated metabolic pathway perturbations, two complementary enrichment strategies were employed. Quantitative enrichment analysis (QEA) identified protein digestion and absorption—primarily involving amino acids and their derivatives—as the most significantly perturbed pathway (Q-statistic = 10.39, FDR = 3.77 × 10^−9^), followed by D-amino acid metabolism (Q-statistic = 6.91, FDR = 5.62 × 10^−6^) and linoleic acid metabolism (Q-statistic = 6.28, FDR = 1.61 × 10^−5^) (Figure 2E). Biosynthesis of unsaturated fatty acids (FDR = 0.0034), tryptophan metabolism (FDR = 0.0030), arachidonic acid metabolism (FDR = 0.0172), and folate biosynthesis (FDR = 0.0095) were also significantly perturbed.

Directional ORA revealed that down-regulated metabolites were predominantly enriched in protein digestion and absorption (FDR = 0.0016), linoleic acid metabolism (FDR = 0.0016), and propanoate metabolism (FDR = 0.0439), whereas up-regulated metabolites were mainly enriched in protein digestion and absorption (FDR = 3.67 × 10^−5^), biosynthesis of unsaturated fatty acids (FDR = 7.87 × 10^−5^), tryptophan metabolism (FDR = 0.0048), arginine biosynthesis (FDR = 0.0142), folate biosynthesis (FDR = 0.0142), and glycine/serine/threonine metabolism (FDR = 0.0235) (Figure 2F).

### 3.3. Cross-Omics Integration and Diagnostic Model Construction

**Figure 3:**
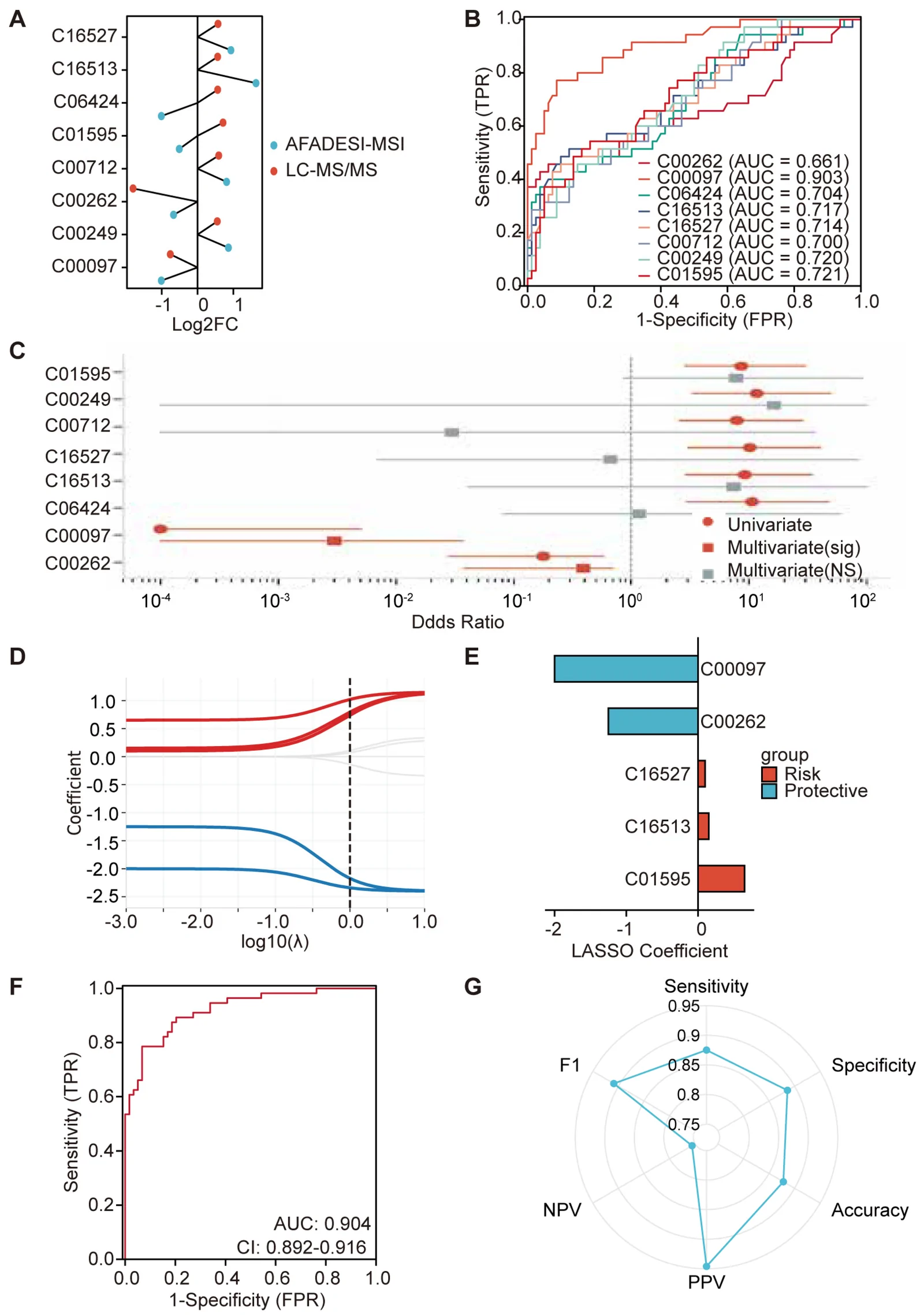
Cross-omics integration and diagnostic model. (**A**) Bidirectional lollipop chart of log_2_ fold changes (FC) of the eight overlapping metabolites across platforms (blue, AFADESI-MSI; red, LC–MS); left, down-regulated; right, up-regulated. (**B**) Univariate ROC curves of the eight metabolites; cysteine (C00097) in red. (**C**) Forest plot of odds ratios (OR) from Firth penalized logistic regression; circles, univariate; squares, multivariate; error bars, 95% confidence intervals (CI); dashed line, OR = 1 (no association); CI > 100 truncated for visualization. (**D**) LASSO coefficient paths; dashed line, optimal λ(log_10_(λ) = 0.0; λ.min selected by the inner 5-fold cross-validation). (**E**) Non-zero coefficients of the five selected metabolites; red, positive association with NPC risk; blue, negative. (**F**) ROC curve of the combined model under leakage-free nested cross-validation; AUC = 0.904 (95% CI: 0.892–0.916); optimal cutoff (0.716) per the Youden index. (**G**) Radar chart of model performance (sensitivity, 0.875; specificity, 0.886; accuracy, 0.878; PPV, 0.946; NPV, 0.756; F1 score, 0.909). NPC, nasopharyngeal carcinoma; AFADESI-MSI, air flow-assisted desorption electrospray ionization mass spectrometry imaging; ROC, receiver operating characteristic; AUC, area under the curve; OR, odds ratio; LC–MS, liquid chromatography–mass spectrometry; LASSO, least absolute shrinkage and selection operator; PPV, positive predictive value; NPV, negative predictive value.

To identify hub metabolites bridging systemic and local metabolic alterations, serum and tissue differential metabolites were intersected based on KEGG compound identifiers, yielding 8 overlapping features: cysteine (C00097), palmitic acid (C00249), hypoxanthine (C00262), oleic acid (C00712), linoleic acid (C01595), myristic acid (C06424), docosapentaenoic acid (DPA, C16513), and adrenic acid (AdA, C16527). Among these, 5 (62.5%) were mapped to the biosynthesis of unsaturated fatty acids pathway (hsa01040). Directional consistency analysis revealed that cysteine (C00097) and hypoxanthine (C00262) were down-regulated in both serum and tissues, whereas DPA (C16513) and AdA (C16527) were up-regulated in both compartments. Palmitic acid (C00249) and oleic acid (C00712) showed concordant trends but with differing magnitudes. Notably, linoleic acid (C01595) and myristic acid (C06424) displayed discordant regulation patterns between the AFADESI-MSI (tissue) and LC–MS (serum) platforms (Figure 3A).

Univariate receiver operating characteristic (ROC) analysis was performed on the 8 overlapping metabolites. Cysteine (C00097) demonstrated the highest discriminatory performance, with an AUC of 0.903 (95% confidence interval (CI): 0.842–0.964) (Figure 3B). Univariate Firth penalized logistic regression showed that all 8 metabolites were significantly associated with NPC risk: cysteine (odds ratio (OR) < 0.001, 95% CI: <0.001–0.005, *p* < 0.001), hypoxanthine (OR = 0.179, 95% CI: 0.028–0.577, *p* < 0.001), myristic acid (OR = 10.602, 95% CI: 2.956–47.077, *p* < 0.001), DPA (OR = 9.195, 95% CI: 2.873–34.189, *p* < 0.001), AdA (OR = 10.123, 95% CI: 3.061–40.008, *p* < 0.001), oleic acid (OR = 7.856, 95% CI: 2.587–28.118, *p* < 0.001), palmitic acid (OR = 11.576, 95% CI: 3.344–48.900, *p* < 0.001), and linoleic acid (OR = 8.586, 95% CI: 2.859–29.850, *p* < 0.001).

Due to the high discriminatory power of cysteine (quasi-complete separation), standard multivariate logistic regression produced unstable maximum likelihood estimates with inflated odds ratios and excessively wide confidence intervals. Therefore, multivariate Firth penalized logistic regression was employed to provide bias-reduced and stable estimates. After Firth penalization, cysteine remained the strongest independent predictor (OR = 0.003, 95% CI: <0.001–0.037, *p* < 0.001), followed by hypoxanthine (OR = 0.390, 95% CI: 0.038–0.694, *p* < 0.001) (Figure 3C). Linoleic acid showed a trend toward independent significance (OR = 7.765, 95% CI: 0.861–90.036, *p* = 0.068). The remaining five metabolites did not retain independent significance in the multivariate model, suggesting that their predictive information was largely captured by cysteine and hypoxanthine.

To construct a combined diagnostic model while avoiding overfitting and information leakage, the entire feature-selection pipeline was embedded within an outer stratified 5-fold cross-validation repeated with 10 random seeds, with an inner 5-fold loop for LASSO–logistic regression. The final model retained five metabolites—cysteine, hypoxanthine, DPA, AdA, and linoleic acid (Figure 3D,E)—and achieved an AUC of 0.904 (95% CI: 0.892–0.916) (Figure 3F). At the optimal cutoff (0.716, Youden index), the model achieved a sensitivity of 0.875, specificity of 0.886, accuracy of 0.878, positive predictive value (PPV) of 0.946, negative predictive value (NPV) of 0.756, and F1 score of 0.909 (Figure 3G). Plasma EBV DNA positivity (≥500 copies/mL) was observed in 53 of 80 patients (66.2%) and was significantly associated with advanced T, N, M, and overall stage (all *p* < 0.01; Supplementary Table S1). None of the eight overlapping metabolites differed between EBV DNA-positive and EBV DNA-negative patients (all *p* > 0.05; Figure S1), and this remained true when correlating log_10_-transformed EBV DNA load with each metabolite (all *p* > 0.05), when adjusting for clinical stage (all *p* > 0.05), and within advanced-stage patients (all *p* > 0.05). Moreover, the five-metabolite panel maintained comparable performance in EBV DNA-positive (AUC = 0.899) and EBV DNA-negative (AUC = 0.913) patients versus healthy controls (Figure S2), indicating that the diagnostic metabolites are not confounded by EBV status.

We next examined whether the eight metabolites varied across clinical stages. Their serum levels showed no consistent pattern of stage-dependent change: although the omnibus Kruskal–Wallis test showed raw *p* < 0.05 for cysteine (*p* = 0.012) and oleic acid (*p* = 0.038), and post hoc comparisons revealed isolated pairwise differences (cysteine: I vs. II; oleic acid and palmitic acid: II vs. III and III vs. IVB), none survived Benjamini–Hochberg correction (all q > 0.09), and no monotonic trend was detected (Jonckheere–Terpstra test; Figure S3). The observed pairwise differences were non-monotonic and predominantly involved the small stage II subgroup (*n* = 5), suggesting random fluctuation rather than true stage-dependent progression. Consistent with this stage-independence, the univariate AUCs of the eight metabolites for distinguishing early-stage (I–II) patients (*n* = 24) from healthy controls ranged from 0.691 to 0.905 (cysteine: AUC = 0.905, 95% CI: 0.832–0.979), nearly identical to those of the full cohort, supporting their utility for early NPC detection (Figure S4). Taken together, these findings delineate a compartmentalized metabolic landscape of NPC—local glycerophospholipid remodeling with ferroptosis susceptibility in tumor tissue, systemic amino-acid-related perturbations in serum, and biosynthesis of unsaturated fatty acids as a shared hub—and identify cysteine and the five-metabolite panel as candidate non-invasive diagnostic markers.

## 4. Discussion

In this study, we integrated serum CIL-LC-MS metabolomics with AFADESI-MSI spatial metabolomics to characterize the compartmentalized metabolic landscape of NPC, addressing the long-standing question of how well the circulating metabolome reflects local tumor metabolism. The Discussion below focuses on the biological interpretation of the compartment-specific and shared metabolic alterations, their consistency with existing evidence, and the implications of the candidate diagnostic metabolites for early NPC detection.

Spatial metabolomics analysis suggested global differences in metabolic profiles between NPC tissues and chronically inflamed tissues. Both ORA and GSEA consistently identified glycerophospholipid metabolism as the most significantly perturbed pathway in NPC tissues (NES = −1.99, FDR = 0.0015), with the driving metabolite phosphatidylserine (C02737; PS(18:1/20:2)) exhibiting markedly reduced ion intensity in tumor regions (log_2_FC = −3.29). A recent multi-omics study integrating single-cell transcriptomics, spatial transcriptomics, and spatial metabolomics identified five glycerophospholipid metabolism-related genes—AGPAT3, DGAT2, SLC44A1, AGPAT5, and LPGAT1—as significantly upregulated in NPC and Epstein–Barr virus-encoded small RNA (EBER)^+^ tumor-enriched regions, suggesting that glycerophospholipid metabolism plays an important role in NPC progression [17]. Notably, glycerophospholipid metabolism exhibited opposite enrichment directions between positive- and negative-ion modes (NES = +1.64 vs. −1.99), suggesting active phospholipid turnover in NPC tissues. Cancer cells adapt to microenvironmental demands through phospholipid remodeling [18,19], and CDS1 (CDP-diacylglycerol synthase 1) was significantly downregulated in NPC [20], further supporting the involvement of lipid metabolic remodeling in NPC pathogenesis.

The ferroptosis pathway enrichment in spatial ORA (FDR = 0.0108) is mechanistically informative. Ferroptosis—an iron-dependent, lipid-peroxidation-driven form of regulated cell death—requires two convergent conditions: (i) sufficient polyunsaturated fatty acid (PUFA)-phospholipid substrates for peroxidation and (ii) impaired glutathione peroxidase 4 (GPX4)-mediated lipid peroxide detoxification. Our observation that NPC tissues exhibit both glycerophospholipid remodeling (generating PUFA-enriched phospholipids) and systemic cysteine depletion (limiting glutathione synthesis) suggests that NPC exists in a “ferroptosis-primed” metabolic state. This is consistent with reports that SLC7A11, GCLM, and GCLC—the core machinery of cystine import and glutathione (GSH) biosynthesis—are paradoxically upregulated in NPC yet associated with poor prognosis, possibly reflecting a compensatory response to chronic oxidative stress [21]. Importantly, this primed state does not necessarily imply spontaneous ferroptosis in vivo (given the complexity of tumor microenvironment antioxidant defenses), but rather identifies a metabolic vulnerability exploitable by ferroptosis inducers [22].

In contrast to the local glycerophospholipid remodeling and ferroptosis-related metabolic alterations observed in tumor tissues, serum metabolomics revealed broader systemic metabolic perturbations in NPC patients. PCA and OPLS-DA analyses demonstrated significant separation in metabolic profiles between the NPC and healthy control groups (R^2^Y = 0.916, Q^2^ = 0.837), indicating robust systemic metabolic reprogramming. A total of 323 differential metabolites were identified, involving alterations across multiple pathways including amino acid metabolism, lipid metabolism, and one-carbon metabolism, consistent with previous serum metabolomics studies in NPC [23].

QEA and directional ORA consistently demonstrated systemic activation of amino acid metabolism in NPC serum. Tryptophan metabolism (QEA: FDR = 0.0030; ORA: enriched in the up-regulated metabolite set, FDR = 0.0048), arginine biosynthesis (ORA: FDR = 0.0142), and glycine/serine/threonine metabolism (ORA: FDR = 0.0235) were significantly enriched. These alterations are consistent with the immunosuppressive microenvironment reported in NPC [24,25]. In addition, folate biosynthesis was significantly perturbed in QEA (FDR = 0.0095) and enriched in the up-regulated metabolite set (ORA: FDR = 0.0142), further supporting systemic activation of one-carbon metabolism in NPC [26].

The biosynthesis of unsaturated fatty acids was significantly perturbed in QEA (Q-statistic = 3.49, FDR = 0.0034) and enriched in the up-regulated metabolite set in ORA (FDR = 7.87 × 10^−5^), suggesting systemic activation of fatty acid metabolism in NPC patients. Lipid metabolic reprogramming is considered one of the most prominent metabolic abnormalities in cancer, and NPC cells adapt to harsh environments by enhancing fatty acid uptake, synthesis, and oxidation [8]. Spatially resolved Raman spectroscopy combined with ultra-performance liquid chromatography–tandem mass spectrometry (UPLC-MS/MS) metabolomics confirmed elevated levels of unsaturated fatty acids in NPC cells, particularly in highly metastatic 5-8F cells and poorly differentiated CNE2 cells [27]. Arachidonic acid metabolism was significantly perturbed in QEA (FDR = 0.0172). Linoleic acid metabolism was significantly enriched in the down-regulated metabolite set in ORA (FDR = 0.0016), suggesting a systemic reduction in linoleic acid in NPC serum. Serum metabolomics studies have identified linoleic acid as a key metabolite distinguishing NPC patients from healthy controls [28,29].

Propanoate metabolism was significantly enriched in the down-regulated metabolite set in directional ORA (FDR = 0.0439), suggesting a systemic reduction in propanoate-related metabolites in NPC serum. Short-chain fatty acids (SCFAs) serve not only as energy substrates but also as critical signaling molecules involved in the regulation of intestinal immune homeostasis and systemic inflammatory responses [25]. Integrated 16S rRNA sequencing of tumor tissues and fecal samples combined with targeted metabolomics has confirmed that SCFAs play an important role in the microbiome–metabolite interplay in NPC patients [30].

Chronic inflammation is recognized as a precursor state in nasopharyngeal carcinogenesis [31,32,33], and the chronically inflamed tissue used as the comparator in this study therefore represents a pre-malignant inflammatory state rather than normal tissue. Despite the different comparators used in the tissue and serum analyses, the high directional concordance (6/8) of the overlapping metabolites indicates that their alterations are robust to comparator choice and reflect tumor-intrinsic metabolic reprogramming. Intersection analysis of serum and tissue differential metabolites identified 8 overlapping metabolites, of which 5 (62.5%) belonged to the biosynthesis of unsaturated fatty acids pathway (hsa01040). This pathway was independently enriched in both serum (ORA up-regulated, FDR = 9.33 × 10^−6^) and tissues (negative-ion ORA, FDR = 0.0024), with the key metabolites docosapentaenoic acid (DPA, C16513) and adrenic acid (AdA, C16527) showing consistent up-regulation across both datasets. Spatially resolved Raman spectroscopy combined with UPLC-MS/MS metabolomics confirmed increased levels of unsaturated fatty acids in NPC cells [27]. A systematic analysis based on 21 metabolic pathways further confirmed that biosynthesis of unsaturated fatty acids is a risk factor for NPC [34]. Serum and urine metabolomics studies further revealed that 181 differential metabolites were enriched in 16 pathways, explicitly including the biosynthesis of unsaturated fatty acids pathway [23]. These findings position the biosynthesis of unsaturated fatty acids as a hub pathway bridging systemic and local metabolic alterations.

Directional consistency analysis revealed two discordant metabolites—linoleic acid (C01595, up-regulated in serum but down-regulated in tissue) and myristic acid (C06424, up-regulated in serum but down-regulated in tissue). This systemic–local decoupling phenomenon suggests that certain fatty acids are elevated in the circulation but extensively consumed at the tumor site. Previous studies have demonstrated that fatty acid synthase (FASN) is highly expressed in NPC, closely associated with tumor aggressiveness and poor prognosis [35]. Moreover, linoleic acid has been found to drive tumor progression by activating peroxisome proliferator-activated receptor-α (PPAR-α), which promotes M2-type tumor-associated macrophage infiltration and suppresses NK cell infiltration [36]. Myristic acid, on the other hand, enhances oncogenic signaling through promoting Src kinase myristoylation [37]. Therefore, the elevated circulating levels of linoleic acid and myristic acid may reflect a systemic demand for fatty acids by the tumor, while their reduction at the tumor site likely results from active uptake and utilization by tumor cells to support membrane biosynthesis, energy metabolism, and protein modifications [38]. This finding underscores the limitation of relying solely on serum biomarkers to infer tumor metabolic status and highlights the unique value of spatial metabolomics in revealing tissue-specific metabolic alterations.

Regarding diagnostic biomarkers, cysteine (C00097) demonstrated excellent single-variable diagnostic performance with an AUC of 0.903. Cysteine serves as the rate-limiting precursor for glutathione (GSH) synthesis, which is essential for GPX4-mediated clearance of lipid peroxides. In NPC, high SLC7A11 expression has been confirmed to be significantly associated with poor prognosis, metastasis, and advanced T stage. Targeting SLC7A11 leads to restricted GSH biosynthesis, accumulation of reactive oxygen species, and lipid peroxidation, ultimately inducing ferroptosis in NPC cells [21]. Meanwhile, aberrant activation of the System Xc^−^/GSH/GPX4 antioxidant axis has been established as a core mechanism of radiotherapy resistance in NPC [21]. Overexpression of SLC7A11 and GPX4 has been identified as an independent risk factor for radiotherapy resistance in NPC patients [39]. Systemic cysteine depletion—combined with local accumulation of PUFAs and phospholipid remodeling—suggests that NPC patients exhibit a ferroptosis-primed metabolic state, rendering tumor cells more susceptible to ferroptosis inducers.

LASSO regression selected five metabolites with non-zero coefficients from the eight overlapping metabolites. Under leakage-free nested cross-validation, the combined model achieved an AUC of 0.904 (95% CI: 0.892–0.916). The panel was significantly superior to the best single metabolite, cysteine (DeLong test, *p* < 0.001), although the absolute improvement was modest. A 9-metabolite signature based on serum metabolomics has been validated as a reliable prognostic indicator for progression-free survival in patients with locoregionally advanced NPC [40]. Serum endogenous small-molecule metabolite profiling has also been shown to be useful for NPC diagnosis and chemoradiotherapy response assessment [41]. These findings suggest that combined multi-metabolite models can provide complementary diagnostic information and hold significant potential for clinical translation. Notably, plasma EBV DNA positivity was associated with advanced stage, consistent with its established role as a tumor burden marker [1], whereas EBER positivity reflects viral latency within tumor cells. Importantly, none of the diagnostic metabolites was associated with EBV DNA status, and the five-metabolite panel performed comparably in EBV DNA-positive (AUC = 0.899) and EBV DNA-negative (AUC = 0.913) patients, supporting the applicability of the panel across EBV-defined subgroups. In addition, the diagnostic metabolites showed no consistent stage-dependent change, and their individual AUCs in early-stage (I–II) patients (0.691–0.905) were comparable to those of the full cohort, further supporting their applicability for early NPC detection. Validation in larger early-stage cohorts is warranted.

Several limitations of the present study warrant consideration. First, the spatial metabolomics cohort comprised only three independent biological specimens per group. The specimen-level exact permutation sensitivity analysis yielded no BH-significant features, so the tissue findings remain exploratory and hypothesis-generating; physical tissue sections and MSI pixels do not increase the independent biological sample size. Second, the cross-sectional nature of the study design precludes any causal inferences. Third, the serum and tissue samples were not obtained from the same patient cohorts. Consequently, larger-scale independent cohort studies complemented by functional experiments are required to further corroborate our conclusions.

## 5. Conclusions

In conclusion, integrated CIL-LC-MS serum metabolomics and AFADESI-MSI spatial metabolomics revealed compartmentalized metabolic reprogramming in NPC, characterized by systemic amino acid and propanoate perturbations alongside local glycerophospholipid remodeling and ferroptosis susceptibility. The biosynthesis of unsaturated fatty acids served as a hub pathway bridging systemic and local metabolic alterations. Circulating cysteine (C00097) emerged as a promising non-invasive diagnostic biomarker, and the 5-metabolite combined model demonstrated robust discriminatory performance. These findings position NPC in a ferroptosis-primed metabolic state and highlight the therapeutic potential of targeting the glycerophospholipid-iron axis in this malignancy.

## Figures and Tables

**Table 1:** Baseline characteristics of the tissue specimens.

Sample ID	Gender	Age (Years)	Pathological Diagnosis	TNM Stage	EBER	Group
Sample 1	Male	54	Nonkeratinizing undifferentiated carcinoma	T3N0M0	Positive	NPC
Sample 2	Female	59	Nonkeratinizing undifferentiated carcinoma	T3N3M0	Positive	NPC
Sample 3	Male	42	Nonkeratinizing undifferentiated carcinoma	T4N1M0	Positive	NPC
Sample 4	Female	16	Chronic nasopharyngitis	—	Negative	CI
Sample 5	Female	51	Chronic nasopharyngitis	—	Negative	CI
Sample 6	Male	62	Chronic nasopharyngitis	—	Negative	CI

NPC, nasopharyngeal carcinoma; CI, chronic inflammation; EBER, Epstein–Barr virus-encoded small RNA; TNM, tumor-node-metastasis; AJCC/UICC, American Joint Committee on Cancer/Union for International Cancer Control. TNM staging was performed according to the 8th edition of the AJCC/UICC staging system. EBER positivity was determined by in situ hybridization. —, not applicable.

**Table 2:** Baseline characteristics of participants in the serum metabolomics cohort.

Characteristics	NPC (*n* = 80)	HC (*n* = 35)	*p* Value
Sex, n (%)			0.14
Male	61 (76.3)	22 (62.9)	
Female	19 (23.8)	13 (37.1)	
Age, mean ± SD	50.1 ± 11.6	47.7 ± 6.4	0.16
T stage, n (%)			
T1	22 (27.5)	-	
T2	8 (10.0)	-	
T3	19 (23.8)	-	
T4	31 (38.8)	-	
N stage, n (%)			
N0	24 (30.0)	-	
N1	25 (31.2)	-	
N2	8 (10.0)	-	
N3	23 (28.7)	-	
M stage, n (%)			
M0	56 (70.0)	-	
M1	24 (30.0)	-	
Clinical stage, n (%)			
I	19 (23.8)	-	
II	5 (6.2)	-	
III	8 (10.0)	-	
IVA	24 (30.0)	-	
IVB	24 (30.0)	-	
EBV DNA load, median (IQR)	2285 (196.25, 41,520)	-	
EBV DNA positive, n (%)	53 (66.2)	-	

Data are presented as mean ± SD, n (%), or median (IQR). *p* values were calculated using the unpaired t-test for age and the chi-square test for sex. TNM staging followed the 8th edition of the AJCC/UICC system: T, extent of the primary tumor (T1–T4); N, regional lymph node involvement (N0–N3); M, distant metastasis (M0, absent; M1, present). EBV DNA positivity was defined as plasma EBV DNA ≥ 500 copies/mL. -, not applicable (the HC group had no tumor-related characteristics). NPC, nasopharyngeal carcinoma; HC, healthy control; SD, standard deviation; IQR, interquartile range; EBV, Epstein–Barr virus; TNM, tumor-node-metastasis; AJCC/UICC, American Joint Committee on Cancer/Union for International Cancer Control.

## Data Availability

The data presented in this study are available on request from the corresponding author. The data are not publicly available due to hospital regulations and to protect patient privacy.

## Supplementary Materials

The following supporting information can be downloaded at: https://www.mdpi.com/article/10.3390/metabo16100783/s1, Supplementary Methods S1–S5: detailed experimental and analytical procedures, including reagents and materials, serum sample preparation and CIL-LC-MS protocols, tissue section preparation and AFADESI-MSI parameters, MSI data preprocessing and molecular annotation, and statistical analysis and model validation. Table S1: baseline characteristics of NPC patients stratified by plasma EBV DNA status. Table S2: association between the eight overlapping metabolites and plasma EBV DNA status in the serum cohort (n = 80). Figure S1: serum levels of the five panel metabolites in EBV DNA–positive and EBV DNA–negative NPC patients. Figure S2: diagnostic performance of the five-metabolite panel in EBV-defined subgroups of NPC patients. Figure S3: serum levels of the eight overlapping metabolites across clinical stages in NPC patients. Figure S4: univariate diagnostic performance of the eight overlapping metabolites for early-stage NPC detection.

## Abbreviations

The following abbreviations are used in this manuscript:

AdA	adrenic acid
AFADESI-MSI	air flow-assisted desorption electrospray ionization mass spectrometry imaging
AJCC/UICC	American Joint Committee on Cancer/Union for International Cancer Control
AUC	area under the receiver operating characteristic curve
CI	chronic inflammation
CIL	chemical isotope labeling
DPA	docosapentaenoic acid
EBER	Epstein–Barr virus-encoded small RNA
EBV	Epstein–Barr virus
FASN	fatty acid synthase
FDR	false discovery rate
FC	fold change
GPX4	glutathione peroxidase 4
GSEA	gene set enrichment analysis
GSH	glutathione
H&E	hematoxylin and eosin
HC	healthy control
IQR	interquartile range
KEGG	Kyoto Encyclopedia of Genes and Genomes
LASSO	least absolute shrinkage and selection operator
LC–MS	liquid chromatography–mass spectrometry
LC–MS/MS	liquid chromatography–tandem mass spectrometry
MSI	mass spectrometry imaging
NEG	negative-ion mode
NES	normalized enrichment score
NPC	nasopharyngeal carcinoma
NPV	negative predictive value
OPLS-DA	orthogonal partial least squares discriminant analysis
OR	odds ratio
ORA	over-representation analysis
PCA	principal component analysis
PD-1	programmed cell death protein 1
POS	positive-ion mode
PPAR-α	peroxisome proliferator-activated receptor-α
PPV	positive predictive value
PS	phosphatidylserine
PUFA	polyunsaturated fatty acid
Q-TOF	quadrupole time-of-flight
QEA	quantitative enrichment analysis
ROC	receiver operating characteristic
SCFAs	short-chain fatty acids
SD	standard deviation
TIC	total ion current
TNM	tumor-node-metastasis
TUS	total useful signal
UHPLC	ultra-high-performance liquid chromatography
UMAP	uniform manifold approximation and projection
UPLC-MS/MS	ultra-performance liquid chromatography–tandem mass spectrometry
VIP	variable importance in projection
