# Supplementary material for: Integrated Serum and Spatial Metabolomics Reveals Compartmentalized Metabolic Reprogramming and Ferroptosis Susceptibility in Nasopharyngeal Carcinoma
Source: Metabolites. 2026 Oct 16;16(10):783. doi: 10.3390/metabo16100783 (PMC13706798; doi:10.3390/metabo16100783)
Supplement: Supplementary file 1 [file metabolites-16-00783-s001.zip › metabolites-4596042_Supplementary Methods.pdf]

Supplementary Methods

S1. Reagents and Materials

LC–MS grade methanol, acetonitrile, and formic acid were purchased from Thermo Fisher Scientific (Pittsburgh, PA, USA). Chemical isotope labeling (CIL) kits for amine/phenol metabolites (dansyl chloride labeling) and carboxyl metabolites (DmPA labeling) were provided by the analytical service provider. Cryo-Gel embedding medium for frozen sectioning was obtained from Leica Biosystems (Germany), and positively charged slides were purchased from Epredia (USA).

S2. Detailed Serum Sample Preparation and CIL-LC-MS Protocols

S2.1. Serum Sample Collection and Preparation

Peripheral venous blood was collected from all participants in the early morning (7:00 a.m.) after an overnight fast of at least 10 h using standard vacuum blood collection tubes. Samples were allowed to clot at room temperature for 30 min, followed by centrifugation at 1,500 × g for 5 min at 4°C. The supernatant serum was transferred into 1.5 mL cryotubes, snap-frozen in liquid nitrogen, and stored at −80 °C for 3 months until analysis, with all samples undergoing a single freeze–thaw cycle prior to extraction.

For metabolite extraction, serum samples were thawed on ice and vortexed. Serum (20 μL) was mixed with 90 μL of pre-cooled (−20°C) LC–MS grade methanol. The mixture was vortexed thoroughly, incubated at −20°C for 1 h to precipitate proteins, and centrifuged at 12,000 rpm for 10 min at 4°C. The supernatant (90 μL) was transferred to a new tube and dried under vacuum.

S2.2. Chemical Isotope Labeling (CIL)

Dried metabolite extracts were subjected to two-channel chemical isotope labeling (CIL) according to the manufacturer's standard operating procedures.

Amine/Phenol channel: Dried residues were reconstituted in 25 μL of LC–MS grade water. Labeling was performed by sequential addition of 12.5 μL buffer reagent A and 37.5 μL of ^{12}C-dansyl chloride (Dns) labeling reagent (for individual samples) or ^{13}C-Dns reagent (for the pooled quality control sample). The mixture was vortexed and incubated at 40°C for 45 min. The reaction was quenched by adding 7.5 μL reagent C, followed by incubation at 40°C for 10 min. Finally, 30 μL of pH adjustment reagent D was added.

Carboxyl channel: Dried residues were reconstituted in 25 μL of acetonitrile/water (3:1, v/v). Labeling was performed by adding 10 μL catalytic reagent A and 25 μL of ^{12}C-DmPA labeling reagent (for individual samples) or ^{13}C-DmPA reagent (for the pooled sample). The mixture was vortexed and incubated at 80°C for 60 min, followed by quenching with 40 μL reagent C and incubation at 80°C for 30 min. After labeling, the ^{13}C-labeled pooled sample was quantified by LC-UV and spiked into each ^{12}C-labeled individual sample as an internal standard. A quality control (QC) sample was prepared by mixing equal volumes of ^{12}C- and ^{13}C-labeled pooled samples.

S2.3. LC–MS Conditions

Chromatographic separation was performed on an Agilent 1290 UHPLC system coupled with an Agilent Eclipse Plus C18 column (150 × 2.1 mm, 1.8 μm). Mobile phase A was 0.1% (v/v) formic acid in water, and mobile phase B was 0.1% (v/v) formic acid in acetonitrile. The gradient elution program was: 0 min, 25% B; 10 min, 99% B; 13 min, 99% B; 15 min, 99% B; 15.1 min, 25% B; and 18 min, 25% B. The flow rate was 400 μL/min, and the column temperature was 40°C.

Mass spectrometry detection was performed on an Agilent 6546 Q-TOF mass spectrometer. Data were acquired in full-scan mode over an m/z range of 220–1000. The QC sample and a retention time calibration sample were analyzed every 16 analytical samples to monitor instrument stability and

retention time drift.

S3. Detailed Tissue Section Preparation and AFADESI-MSI Parameters

S3.1. Preparation of Frozen Tissue Sections

Frozen tissue samples were retrieved from −80°C storage and transferred to a cryotome (DAKEWE CT520, China) pre-cooled to −20°C for approximately 30 min to equilibrate. Cryo-Gel embedding medium was applied to the specimen holder, and the frozen nasopharyngeal tissue was placed on the embedding medium oriented to expose the maximum cross-sectional plane. After positioning, additional embedding medium was applied to completely cover the tissue, and the specimen was left at −20°C for approximately 30 min to ensure full solidification. Following embedding, serial 12-μm-thick sections were cut using the cryotome and mounted onto pre-chilled positively charged slides, then stored at −80°C until further use.

S3.2. AFADESI-MSI Mass Spectrometry Imaging

Air-flow-assisted desorption electrospray ionization mass spectrometry imaging (AFADESI-MSI) was performed as previously described [15], with minor modifications. AFADESI-MSI data acquisition was conducted on a platform coupled to a Q-Exactive Orbitrap mass spectrometer (Thermo Scientific, Bremen, Germany). The spray solvent consisted of acetonitrile/water (80:20, v/v), delivered at a flow rate of 5.0 μL/min. Nitrogen (N_2) was used as the nebulizing gas, with a spray pressure of 0.65 MPa and an extraction gas flow rate of 80 L/min. Data were acquired in both positive- and negative-ion modes, with spray voltages set at +4500 V and −4500 V, respectively. The sprayer-to-sample distance and the sprayer-to-ion transfer tube distance were both set to 3 mm. The mass resolution was set at 70,000, with a mass acquisition range of m/z 70–1000. The automatic gain control (AGC) target was set at 5×10^5, with a maximum injection time of 300 ms and a capillary temperature of 350°C. A spatial resolution of 50 μm × 50 μm was achieved. To minimize batch effects, NPC and inflamed tissue samples were mounted on the same glass slide and analyzed in a single AFADESI-MSI run.

S4. Detailed MSI Data Preprocessing and Molecular Annotation

Raw MSI data were converted to mzML format using MSConvertGUI (ProteoWizard 3.0), and subsequently to imzML format using imzMLConverter. The converted data were imported into the MATLAB toolbox MSiToolbox for further processing. Single-dataset preprocessing included denoising, peak alignment, total ion current (TIC) normalization, and low-quality feature filtering. The 10th percentile of the peak intensity distribution was used as a threshold to remove low-intensity m/z features. Peak alignment was performed within a mass tolerance of ±5 ppm, and features with a detection frequency of less than 1% across all tissue-associated pixels were filtered out. K-means clustering was performed with K = 2, based on the prior knowledge that each tissue section comprises two distinct regions: background and tissue. The two resulting clusters were assigned to background and tissue-associated pixels, respectively, and only pixels within tissue regions were retained for subsequent analysis. Following single-dataset preprocessing, cross-sample peak alignment was performed across the six samples to construct a unified feature matrix, followed by TIC- and median-based normalization. Ionic features were further retained if they were detected in at least 2/3 of technical replicates (serial sections) and at least 2/3 of samples within the same group, yielding the final MSI feature matrix. Molecular annotation was facilitated by LC-MS/MS analysis of homologous tissue samples, with the identification results serving as a candidate molecular library. MSI features were matched to the candidate library within a mass tolerance of ±10 ppm. Adduct forms considered in positive-ion mode included $[M+H]^+$, $[M+Na]^+$, and $[M+K]^+$, while $[M-H]^-$ was considered in negative-ion mode.

S5. Detailed Statistical Analysis and Model Validation

S5.1. Dimensionality Reduction and Differential Analysis of Spatial Metabolomics Data

Following data preprocessing, Uniform Manifold Approximation and Projection (UMAP) was applied to the normalized MSI feature matrix for nonlinear dimensionality reduction (Python umap-learn package, n_neighbors=30, min_dist=0.3, Euclidean distance, with a fixed random seed for reproducibility). Data from positive- and negative-ion modes were processed separately and integrated at the annotated compound level. MSI features with a non-zero detection rate of less than 10% in the combined two groups were excluded from subsequent analyses. The exploratory pixel-level spatial screen used Welch's t-test with Benjamini–Hochberg correction. Features meeting both the pixel-level FDR criterion (FDR < 0.05) and the fold-change criterion (FC > 1.2 or FC < 0.83) were retained as exploratory spatial candidates; pixel-level P values were not interpreted as evidence from independent biological replicates. Over-representation analysis (ORA) was performed using a hypergeometric test to map differentially abundant metabolites to KEGG pathways, with FDR < 0.05 considered the threshold for significant enrichment. Pre-ranked gene set enrichment analysis (GSEA) was performed based on $\log_2$FC-ranked lists of all annotated metabolites for positive- and negative-ion mode data separately. GSEA was performed using the R package fgsea (v1.20.0), employing the normalized enrichment score (NES) to evaluate the direction and magnitude of pathway enrichment. FDR < 0.05 was considered significant, and 0.05 < FDR < 0.25 was considered suggestive enrichment.

The independent biological unit was the biological specimen (three NPC and three chronic-inflammation specimens). Each specimen contributed three physical tissue sections, which were within-specimen repeated observations; MSI pixels were nested within sections. For the biological-specimen-level sensitivity analysis, feature intensities were first averaged within each section, and the three section means were then averaged with equal weight to obtain one value per specimen. A two-sided exact permutation test used the absolute difference between the NPC and chronic-inflammation group means and enumerated all 20 possible 3-versus-3 label allocations. Benjamini–Hochberg correction was applied separately within each ion mode. The minimum attainable exact P value was 0.10, and no feature was significant after correction. The tissue findings were therefore interpreted as exploratory and hypothesis-generating.

S5.2. Statistical Analysis and Pathway Enrichment of Serum Metabolomics Data

Differential metabolites were identified using Welch's t-test combined with Benjamini–Hochberg multiple testing correction. The selection criteria were: (i) variable importance in projection (VIP) score > 1.0 from OPLS-DA, indicating strong discriminatory power; (ii) false discovery rate (FDR) < 0.05, ensuring statistical significance after multiple testing correction; and (iii) absolute fold change (|FC|) > 1.2. The |FC| > 1.2 threshold was deliberately chosen because metabolites typically exhibit smaller fold changes compared to proteins or transcripts due to tighter metabolic regulation and homeostatic control, and the high quantitative accuracy of CIL-LC-MS with isotope-labeled internal standards (CV < 15%) enables confident detection of moderate metabolic perturbations that might be obscured in conventional label-free workflows. Over-representation analysis (ORA) was performed using a hypergeometric test to map differentially abundant metabolites to KEGG pathways, with differential metabolites divided into up-regulated and down-regulated sets for separate enrichment analyses to determine the directionality of pathway regulation (activation or inhibition).

Quantitative enrichment analysis (QEA) was performed using MetaboAnalyst 5.0 to assess overall pathway perturbations based on the quantitative information of all detected metabolites. Both QEA and ORA were considered significant at FDR < 0.05.

S5.3. Cross-Omics Integration and Diagnostic Model Construction

Serum and tissue differential metabolites were intersected based on KEGG compound identifiers (IDs) to obtain cross-omics overlapping metabolites, and the concordance of log_2FC directionality between compartments was evaluated. Univariate ROC analysis was performed using the R package pROC (v1.18.0). Univariate and multivariate Firth penalized logistic regression (R package logistf, v1.26.0) was used to evaluate metabolite–NPC associations.

To avoid information leakage, the entire feature-selection pipeline was embedded within an outer stratified 5-fold cross-validation, repeated with 10 random seeds. Within each training partition: (i) serum differential metabolites were re-identified using training samples only (Welch's t-test with Benjamini–Hochberg FDR < 0.05 and |FC| > 1.2, together with VIP > 1.0 from OPLS-DA fitted on the training partition); (ii) the resulting list was intersected with the tissue-derived differential metabolite list (from the independent tissue cohort); and (iii) candidate metabolites were modeled by LASSO–logistic regression (R package glmnet, v4.1.4; λ.min via an inner 5-fold cross-validation) constructed on the training partition only. Each held-out fold was predicted by the corresponding training-fold model. Pooled predicted probabilities across outer-loop test sets were used to construct ROC curves and to compute the AUC, sensitivity, specificity, accuracy, PPV, NPV, and F1 score at the optimal Youden-index cutoff. Subgroup performance (EBV DNA status, clinical stage) was evaluated using the same leakage-free pipeline. All statistical analyses were performed using R 4.2.0.
