# Supplementary material for: Integrated Serum and Spatial Metabolomics Reveals Compartmentalized Metabolic Reprogramming and Ferroptosis Susceptibility in Nasopharyngeal Carcinoma
Source: Metabolites. 2026 Oct 16;16(10):783. doi: 10.3390/metabo16100783 (PMC13706798; doi:10.3390/metabo16100783)
Supplement: Supplementary file 1 [file metabolites-16-00783-s001.zip › metabolites-4596042_supplementary.pdf]

Supplementary Table S1. Baseline characteristics of NPC patients stratified by plasma EBV DNA status.

Characteristic	EBV DNA–negative (n = 27)	EBV DNA–positive (n = 53)	P value
Age, years, mean ± SD	47.2 ± 9.4	51.6 ± 12.4	0.084
Male sex, n (%)	20 (74.1)	41 (77.4)	0.961
T stage, n (%)			<0.001
T1	16 (59.3)	6 (11.3)	
T2	2 (7.4)	6 (11.3)	
T3	4 (14.8)	15 (28.3)	
T4	5 (18.5)	26 (49.1)	
N stage, n (%)			<0.001
N0	17 (63.0)	7 (13.2)	
N1	8 (29.6)	17 (32.1)	
N2	1 (3.7)	7 (13.2)	
N3	1 (3.7)	22 (41.5)	
M stage, n (%)			0.01
M0	24 (88.9)	32 (60.4)	
M1	3 (11.1)	21 (39.6)	
Clinical stage, n (%)			<0.001
I	16 (59.3)	3 (5.7)	
II	1 (3.7)	4 (7.5)	
III	3 (11.1)	5 (9.4)	
IVA	4 (14.8)	20 (37.7)	
IVB	3 (11.1)	21 (39.6)	

EBV DNA positivity was defined as plasma EBV DNA ⩾ 500 copies/mL. P values were calculated using the unpaired t-test (age), chi-square test (sex), and chi-square test with Fisher's exact correction where appropriate (T, N, M, and clinical stage). NPC, nasopharyngeal carcinoma; EBV, Epstein–Barr virus; SD, standard deviation.

Table S2. Association between the eight overlapping metabolites and plasma EBV DNA status in the serum cohort (n = 80).

Metabolite	EBV+ vs EB− P	Spearman ρ	Spearman P	Stage-adjusted P[a]	Advanced-stage P[b]
Cysteine	0.699	0.049	0.664	0.993	0.623
Hypoxanthine	0.436	0.133	0.24	0.665	0.299
Myristic acid	0.718	−0.018	0.875	0.691	0.684
Palmitic acid	0.576	0.015	0.893	0.373	0.615
Oleic acid	0.923	−0.032	0.78	0.491	0.661
Linoleic acid	0.827	−0.135	0.233	0.879	0.864
DPA	0.899	−0.075	0.507	0.466	0.864
AdA	0.47	0.021	0.851	0.4	0.839

EBV DNA positivity was defined as plasma EBV DNA ⩾ 500 copies/mL. Group comparisons were

performed using the Wilcoxon rank-sum test. Spearman correlation (ρ) was computed between $\log_{10}$-transformed plasma EBV DNA load and metabolite intensity.a:Partial Spearman correlation adjusting for clinical stage (ordinal: I = 1, II = 2, III = 3, IVA = 4, IVB = 5). b:Wilcoxon test restricted to stage III – IVB patients (n = 56). Benjamini – Hochberg false discovery rate (FDR) correction was applied within each analysis family of eight metabolites (all FDR-adjusted q values > 0.86); no comparison reached statistical significance (all P > 0.05). DPA, docosapentaenoic acid; AdA, adrenic acid; EBV, Epstein – Barr virus.

Figure S1. Serum levels of the five panel metabolites in EBV DNA–positive and EBV DNA–negative NPC patients.

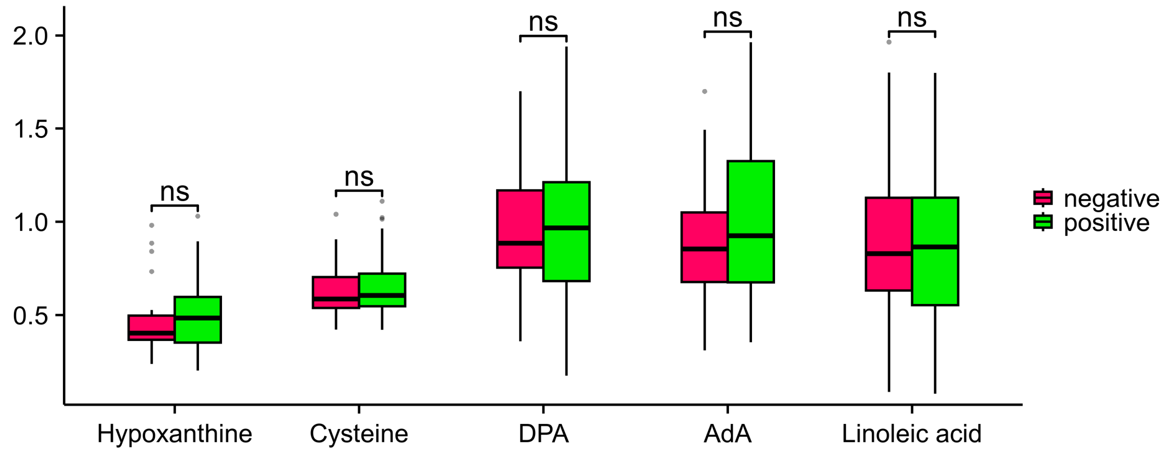

Box-and-whisker plots of the relative serum abundances of the five panel metabolites—hypoxanthine (C00262), cysteine (C00097), docosapentaenoic acid (DPA, C16513), adrenic acid (AdA, C16527), and linoleic acid (C01595, from top to bottom)—in EBV DNA – positive (n = 53) and EBV DNA – negative (n = 27) NPC patients. Boxes show median and interquartile range; whiskers show minimum and maximum. Group comparisons were performed using the Wilcoxon rank-sum test; no comparison was statistically significant (all P > 0.05, indicated as ns). NPC, nasopharyngeal carcinoma; EBV, Epstein – Barr virus; ns, not significant.

Figure S2. Diagnostic performance of the five-metabolite panel in EBV-defined subgroups of NPC patients.

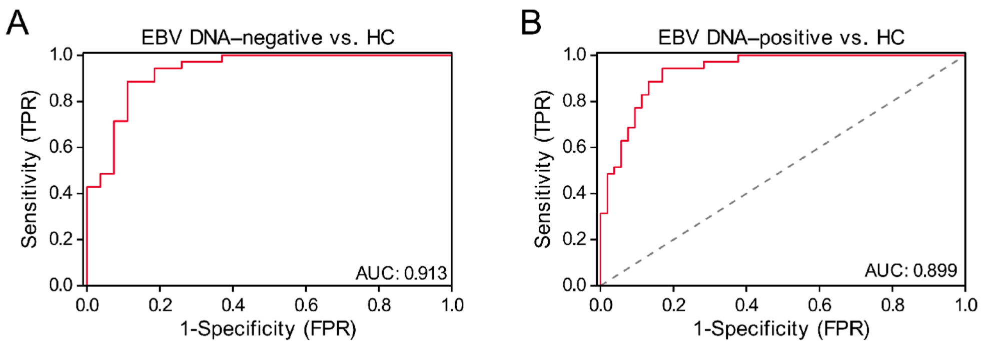

Receiver operating characteristic (ROC) curves of the five-metabolite panel (cysteine, hypoxanthine, DPA, AdA, and linoleic acid) for distinguishing EBV-defined patient subgroups from healthy controls (n = 35). (a) EBV DNA–negative patients (n = 27; area under the curve (AUC) = 0.913); (b) EBV DNA–positive patients (n = 53; AUC = 0.899). Predicted probabilities were derived from leakage-free nested cross-validation as described in the Methods. ROC, receiver operating characteristic; AUC, area under the curve; EBV, Epstein–Barr virus; NPC, nasopharyngeal carcinoma.

Figure S3. Serum levels of the eight overlapping metabolites across clinical stages in NPC patients.

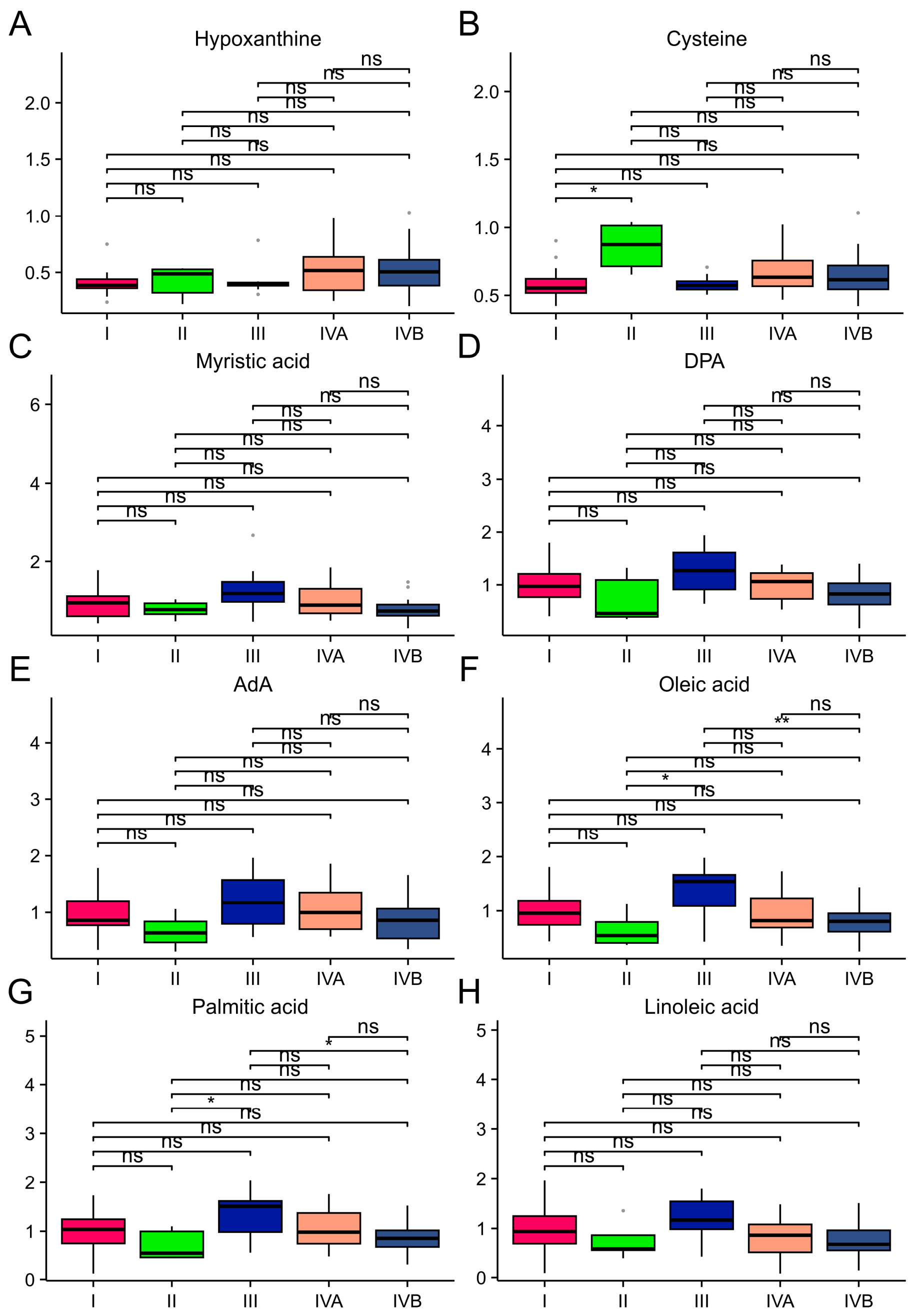

(A–H) Relative serum abundances of hypoxanthine (A), cysteine (B), myristic acid (C), DPA (D), AdA (E), oleic acid (F), palmitic acid (G), and linoleic acid (H) in NPC patients stratified by clinical stage (I, n = 19; II, n = 5; III, n = 8; IVA, n = 24; IVB, n = 24). Boxes show median and interquartile range; whiskers show minimum and maximum. Group differences were assessed by the Kruskal–Wallis test with Dunn's multiple comparisons test (*P < 0.05, **P < 0.01, ns, not

significant). NPC, nasopharyngeal carcinoma; DPA, docosapentaenoic acid; AdA, adrenic acid; ns, not significant.

Figure S4. Univariate diagnostic performance of the eight overlapping metabolites for early-stage NPC detection.

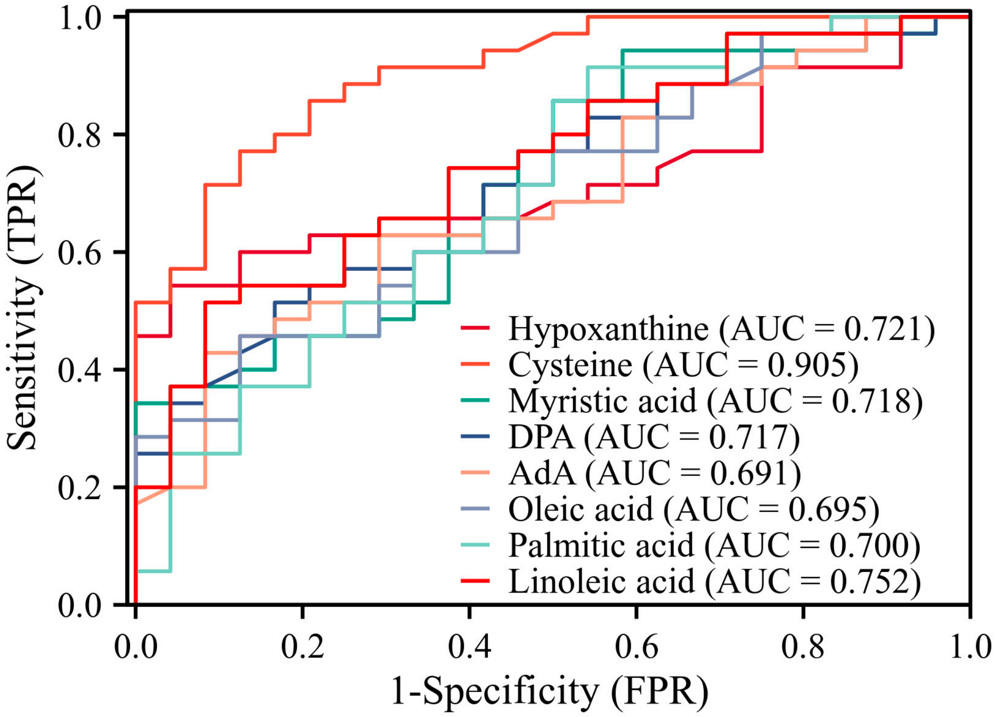

Receiver operating characteristic (ROC) curves of the eight overlapping metabolites for distinguishing early-stage (I – II) NPC patients (n = 24) from healthy controls (n = 35). Cysteine (C00097) achieved the highest discriminatory performance (AUC = 0.905, 95% CI: 0.832 – 0.979, red curve), followed by linoleic acid (C01595; AUC = 0.752, 95% CI: 0.629 – 0.876), hypoxanthine (C00262; AUC = 0.721, 95% CI: 0.591 – 0.852), myristic acid (C06424; AUC = 0.718, 95% CI: 0.585 – 0.850), DPA (C16513; AUC = 0.717, 95% CI: 0.586 – 0.847), palmitic acid (C00249; AUC = 0.700, 95% CI: 0.560 – 0.840), oleic acid (C00712; AUC = 0.695, 95% CI: 0.560 – 0.829), and adrenic acid (AdA,

C16527; AUC = 0.691, 95% CI: 0.555 – 0.827). The dashed diagonal line indicates the reference line (AUC = 0.5). NPC, nasopharyngeal carcinoma; AUC, area under the curve; ROC, receiver operating characteristic; DPA, docosapentaenoic acid; AdA, adrenic acid; CI, confidence interval.
